# Physical activity, brain tissue microstructure, and cognition in older adults

**DOI:** 10.1371/journal.pone.0253484

**Published:** 2021-07-07

**Authors:** Robert J. Dawe, Lei Yu, Sue E. Leurgans, Bryan D. James, Victoria N. Poole, Konstantinos Arfanakis, Julie A. Schneider, David A. Bennett, Aron S. Buchman

**Affiliations:** 1 Rush Alzheimer’s Disease Center, Rush University Medical Center, Chicago, IL, United States of America; 2 Department of Diagnostic Radiology and Nuclear Medicine, Rush University Medical Center, Chicago, IL, United States of America; 3 Department of Neurological Sciences, Rush University Medical Center, Chicago, IL, United States of America; 4 Department of Internal Medicine, Rush University Medical Center, Chicago, IL, United States of America; 5 Department of Orthopedic Surgery, Rush University Medical Center, Chicago, IL, United States of America; 6 Department of Biomedical Engineering, Illinois Institute of Technology, Chicago, IL, United States of America; 7 Department of Pathology, Rush University Medical Center, Chicago, IL, United States of America; Nathan S Kline Institute, UNITED STATES

## Abstract

**Objective:**

To test whether postmortem MRI captures brain tissue characteristics that mediate the association between physical activity and cognition in older adults.

**Methods:**

Participants (N = 318) were older adults from the Rush Memory and Aging Project who wore a device to quantify physical activity and also underwent detailed cognitive and motor testing. Following death, cerebral hemispheres underwent MRI to quantify the transverse relaxation rate R_2_, a metric related to tissue microstructure. For analyses, we reduced the dimensionality of the R_2_ maps from approximately 500,000 voxels to 30 components using spatial independent component analysis (ICA). Via path analysis, we examined whether these R_2_ components attenuated the association between physical activity and cognition, controlling for motor abilities and indices of common brain pathologies.

**Results:**

Two of the 30 R_2_ components were associated with both total daily physical activity and global cognition assessed proximate to death. We visualized these components by highlighting the clusters of voxels whose R_2_ values contributed most strongly to each. One of these spatial signatures spanned periventricular white matter and hippocampus, while the other encompassed white matter of the occipital lobe. These two R_2_ components partially mediated the association between physical activity and cognition, accounting for 12.7% of the relationship (p = .01). This mediation remained evident after controlling for motor abilities and neurodegenerative and vascular brain pathologies.

**Conclusion:**

The association between physically activity and cognition in older adults is partially accounted for by MRI-based signatures of brain tissue microstructure. Further studies are needed to elucidate the molecular mechanisms underlying this pathway.

## Introduction

In the absence of treatments to prevent or reduce the symptoms of Alzheimer’s and other forms of related dementia, intensified efforts are now underway to identify modifiable lifestyle behaviors that help to preserve cognition in older adults [1,2]. Notably, many observational studies have reported associations between habitual physical activity level and cognitive levels [3,4] and trajectories [5,6] among older adults. However, the neurobiologic basis of these associations remains incompletely understood, as underscored by our recent work showing that physical activity and cognition are not linked via indices of brain pathologies that are common among older adults [7]. This knowledge gap has impeded the translation of findings from observational studies into effective activity-based interventions against cognitive decline, thus highlighting the importance of identifying factors other than traditional histopathologic indices of brain pathologies that may connect physical activity and cognition.

One avenue for investigation lies in the hypothesis that cognitive benefits of physical activity are related to changes in brain tissue microstructure that can be detected using MRI [8]. This view draws support from animal [9,10] and human studies [11,12] showing that higher levels of habitual physical activity are linked to better white matter integrity on MRI, combined with complementary investigations showing that MR-visible white matter abnormalities are in turn related to impaired cognition [13,14]. Adding further support for white matter microstructure as a potential physiologic mediator of the relation between physical activity and cognition are studies showing that MRI metrics of white matter microstructure and connectivity are related to established correlates of resilience to cognitive decline such as intelligence and to resilience itself [15,16]. The implication of these studies is that healthier white matter reflects a higher degree of recruitment of neural resources and is thus related to heightened brain reserve and maintenance among the crucial networks underlying cognition [17]. However, few studies have examined the interplay of physical activity, white matter microstructure, and cognition in the same well-characterized older individuals [8,18–20]. To address this gap, we used clinical and postmortem data collected in the Rush Memory and Aging Project (MAP), a community-based cohort study of older adults, to examine whether the transverse relaxation rate R_2_, an MRI index related to brain tissue microstructure, mediates the relation between physical activity and cognition in old age.

## Methods

### Participants

MAP is an ongoing cohort study of older adults who agree to annual clinical assessments and brain donation at death [21]. MAP began in 1997 and multiday actigraphy recordings were added to the testing protocol in 2005. Because the current analyses aimed to examine the role of MRI indices of brain tissue microstructure and postmortem indices of neuropathology in the relation between physical activity and cognition, we used data from 624 MAP participants who wore the actigraphy device and died within five years of doing so. Of these cases, 595 participants had valid measures of cognitive function and motor abilities from the same follow-up cycle as the last valid actigraphy recording, and 508 came to autopsy. Postmortem brain imaging was introduced in MAP in 2006. Scanning capacity was expanded over the course of several years, resulting in 318 of the 508 autopsied cases having valid MRI measures of the transverse relaxation rate constant, R_2_, described in more detail below. At enrollment, each MAP participant provided their sex, total years of education, and date of birth. The date of death is known for each case that comes to autopsy, allowing for calculation of age at death.

### Assessment of total daily physical activity

MAP participants wore a watch-like activity monitor continuously for up to 10 consecutive days. An omnidirectional accelerometer within this device generated a signal proportional to the acceleration of the non-dominant wrist, which was rectified and integrated over each 15-second epoch, then saved to onboard memory as an activity count value. We computed the mean total daily physical activity metric by averaging over all days of complete data as described in prior publications [22]. Other investigators have estimated that walking at 2.5 mph results in 2,354 activity counts per minute and jogging at 4.5 mph produces 8,640 activity counts per minute, on average [23]. Thus, an hour of walking or 16 minutes of jogging each contribute roughly 1.4 × 10^5^ counts to the total daily physical activity measure. In these analyses, we used the last available measure of total daily physical activity.

### Assessment of cognition and clinical diagnosis

Participants underwent structured motor and cognitive testing at study entry and during annual follow-up. Trained staff administered a battery of 19 cognitive tests to each participant at annual study visits. Scores on 17 of these tests were normalized based on the mean and standard deviation of the MAP cohort at baseline. The resulting z-scores were then combined to form a composite global cognition score, which has been validated as a sensitive measure of cognitive function and is associated with Alzheimer’s disease pathology and other types of neuropathology in community-dwelling older adults [24,25]. We similarly composed scores for 5 cognitive domains based on different sub-groupings of the 17 tests, based on a previously published factor analysis [26]. These domains and the types of tests from which they are formed are episodic memory (immediate and delayed recall of two stories, word list memory, recall, and recognition), semantic memory (Boston naming, category fluency, and a reading test), working memory (digit span forward and backward and digit ordering tests), perceptual speed (number comparison, Symbol Digit Modalities, and two indices from a modified version of the Stroop Neuropsychological Screening Test), and visuospatial ability (line orientation and progressive matrices tests) [27]. In these analyses, we examined the cognitive scores from the same year as the last actigraphy recording prior to death.

Clinical classification of dementia and cognitive impairment was carried out annually via a uniform process implemented at the inception of MAP. Briefly, an experienced neurologist reviewed data from the aforementioned detailed cognitive testing, a neuropsychologist’s impairment ratings, medical history, and results of neurologic examination and rendered a decision on the presence and likely cause of dementia. Diagnosis of Alzheimer’s dementia was based on the criteria of the joint working group of the National Institute of Neurological and Communicative Disorders and Stroke and the Alzheimer’s Disease and Related Disorders Association (NINCDS/ADRDA) [28,29]. These criteria require a history of cognitive decline as well as impairment in memory and at least one other cognitive domain. Cases of cognitive impairment not meeting the criteria for dementia were classified as mild cognitive impairment (MCI) [21,29].

### Assessment of motor abilities

A composite score of motor abilities was formed from ten measures of eight motor performances, as previously described [30]. These measures include manual dexterity (Purdue Pegboard and finger-tapping speed), time and steps to walk 8 feet, time and steps to turn 360 degrees in place, balancing on toes and on one leg (number of seconds for each, maximum 20), and grip strength and pinch strength quantified via dynamometer. Individual measures were scaled based on the MAP baseline mean, then averaged together to form the summary measure of motor abilities. Although additional tests would help to probe diverse facets of motor abilities with greater accuracy and granularity, the extent of motor testing was somewhat constrained by limits on participant burden in terms of time and exertion, which are imposed in the interest of safety and participant retention in MAP. In these analyses, we used the motor score from the same year as the last actigraphy recording prior to death and normalized this value based on the mean and standard deviation of the sample.

### Assessment of brain tissue microstructure (R_2_) with postmortem MRI

Postmortem MRI of brain tissue offers certain advantages over antemortem MRI, including a reduced consent bias and the opportunity to link each imaging dataset with histopathologic indices from the same specimen almost immediately. Tissue handling and postmortem MRI acquisition procedures have been previously described [31]. At death, the brain was removed and hemisected. One cerebral hemisphere was immersed in 4% paraformaldehyde solution and refrigerated at 4 degrees Celsius. At approximately 30 days postmortem, we imaged the hemisphere using one of four 3-Tesla MRI scanners employed during this ongoing study. We allowed the specimens to warm to room temperature of approximately 20°C before beginning scanning, as previously described [32]. Specimens remained immersed in paraformaldehyde solution during scanning, and we confirmed that the temperature of this surrounding fluid did not change by more than 1°C during the imaging session.

The one-hour scan session included, among other sequences, a 30-minute turbo spin echo sequence with multiple echoes and native resolution of 0.625 mm × 0.625 mm × 1.5 mm, from which we generated R_2_ maps, as previously described [33]. R_2_ is affected by the tissue’s myelin content [34] and may also be sensitive to other tissue characteristics, such as the pathologic accumulation of paramagnetic compounds like iron [35]. While there are a wide range of metrics available in antemortem brain MRI, changes to the tissue after death and fixation mean techniques such as diffusion tensor imaging are not as straightforward to carry out in postmortem brain specimens. R_2_ is the most established postmortem measure in our studies and arguably the field in general. In estimating R_2_, we did not account for the Rician noise characteristics of the MRI signal because the minimum signal-to-noise ratio of the images was approximately 6, which meets the threshold at which Rician noise approximates Gaussian noise [36]. We spatially matched the R_2_ maps to a custom postmortem brain hemisphere template (1-mm isotropic resolution) using first linear and then nonlinear registration with ANTS [37]. Four different MRI scanners were used because the accrual of brain specimens spanned nearly 15 years, during which time three scanners were decommissioned. In combining the data in for analyses, it was therefore necessary to account for inter-instrumental variation. To do so, for each voxel we estimated the mean and standard deviation of R_2_ within the subsamples of specimens imaged using each of the four scanners. We then used these values to transform the raw R_2_ values to normalized z-scores, as in prior work [38].

Due to the large number of voxels in this dataset, voxelwise analyses could possibly yield an excessive number of false positives or negatives, depending on how strictly multiple comparisons are accounted for among the approximately 500,000 voxels. We mitigated this problem by reducing the dimensionality of imaging data using an unsupervised approach, i.e. without regard to any outcome, such as cognition. Specifically, we adapted FSL’s ‘melodic’ tool to carry out spatial independent component analysis (ICA) [39] on the imaging data. Using this method, we identified voxels whose R_2_ values varied together in a similar manner across different specimens. Such variations in R_2_ are thought to reflect commonalities in the tissue’s composition (e.g. myelination or water content), which in turn might arise from shared function, perfusion territory, or other characteristics among those voxels. Pre-processing steps included minor smoothing of the normalized R_2_ maps using FSL’s ‘susan’ tool [40] (smoothing half-width of 1.25 mm) and creation of a mask defining the voxels whose R_2_ values were nonzero (i.e. tissue was present) in that location across all specimens. We set the number of ICs to 30 because 30 ICs captured more than half (55%) of the variance while still providing reasonable reduction of the data into a smaller number of dimensions. This number is consistent with the number of components utilized in several prior brain imaging studies that employed a range of imaging modalities [41–44]. The mixing matrix returned by ICA contained the IC weights, or loadings, which reflected the influence of each IC in the composition of each individual R_2_ map. We used these IC values in further analyses [45].

### Assessment of brain neuropathology

Following postmortem MRI, specimens underwent tissue sectioning and a standard protocol for gross and microscopic examination to compose 10 indices of neurodegenerative and vascular brain pathologies as described in prior work. Briefly, AD pathology was summarized as the average scaled counts of neuritic and diffuse plaques and neurofibrillary tangles in sections from the hippocampus and four cortical regions [46]. We graded nigral neuronal loss semi-quantitatively (none, mild, moderate, severe) [47]. Lewy body pathology was coded as present or absent based on sections from the substantia nigra and six cortical regions [48]. Hippocampal sclerosis was also coded as present or absent [49]. TAR DNA-binding protein 43 (TDP-43) was graded semi-quantitatively based on whether it was absent, appeared only in the amygdala, or also extended to the hippocampus and neocortex [49]. Of the vascular pathologies, chronic gross and microscopic infarcts were each coded as present or absent [50,51]. Each of cerebral atherosclerosis [52], arteriolosclerosis [53], and cerebral amyloid angiopathy [54] were scored semi-quantitatively on four-level scales.

### Analyses

We first examined the bivariate associations of total daily physical activity with age, education, and other participant characteristics using Pearson correlation coefficients. We used *t*-tests to determine whether physical activity differed between males and females.

We then assessed the degree to which postmortem MRI metrics of brain tissue microstructure (R_2_ IC values) might mediate the association between total daily physical activity and global cognition proximate to death, controlling for age at death, sex, and education throughout all analyses. To do so, we employed a two-stage process. In the first stage, we screened for candidate mediators among the 30 R_2_ IC values. This was done by first running 30 separate linear regression models with each of the IC values as predictors and global cognition as the outcome:

$$global\;cognition=\beta_{0}+\beta_{1}*age+\beta_{2}*sex+\beta_{3}*education+\beta_{4}*R_{2,i}$$

where the β terms are the model coefficients to be estimated and R_2,*i*_ represents each of the 30 R_2_ ICs as *i* is incremented from 1 to 30. Also as part of the first screening stage, we ran models with physical activity as the predictor and each of the 30 R_2_ ICs as an outcome:

$$R_{2,i}=\beta_{0}+\beta_{1}*age+\beta_{2}*sex+\beta_{3}*education+\beta_{4}*physical\;activity$$

The R_2_ ICs that were associated with both cognition and physical activity according to this screening procedure were then included as potential mediators in the second stage of analysis, which consisted of path analysis with physical activity as the predictor and cognition as the outcome, again controlling for age, sex, and education. We computed the direct effect, or the unmediated linkage between physical activity and cognition, as well as the indirect effect, which quantifies the potential mediating influence of intermediate variables, in this case the R_2_ ICs (see Fig 3 in Results for visual depiction of the path analysis). We repeated the path analysis with each of the five cognitive domains substituted as the outcome.

We conducted several follow-up analyses to examine the potential influence of a variety of factors on the relationships among physical activity, R_2_, and cognition. First we repeated the path analysis in a stratified manner to explore whether the observed relationships were more prominent in participants who were classified as likely having AD dementia at any point compared to those who either exhibited no cognitive impairments or were classified as having mild cognitive impairment (MCI). Motor abilities are mildly associated with total daily physical activity in the MAP cohort, and thus might also play a role in the relationships among physical activity, brain tissue microstructure (R_2_), and cognition [55]. Therefore, we carried out the path analysis described above with global cognition as the outcome, but also controlled for motor abilities. Similarly, the R_2_ measures may be partially driven by brain pathology [33]. Therefore, we repeated the path analysis while controlling for each of 10 validated indices of neurodegenerative and cerebrovascular brain pathologies, first individually and then together in a combined analysis. Other factors may confound the relationships among physical activity, brain R_2_ measures, and cognition, and we controlled for several such variables that are collected in MAP, including chronic medical conditions, vascular disease and risk factors, depression, and body mass index [21]. To determine whether long intervals between actigraphic data collection and autopsy affected the associations of R_2_ with physical activity and cognition, we carried out a sensitivity analysis in which we restricted the sample to cases for which this interval was less than 2 years.

### Standard protocol approvals, registrations, and patient consents

The Institutional Review Board of Rush University Medical Center approved this study. Participants provided written informed consent and also signed an Anatomical Gift Act for organ donation.

## Results

### Characteristics of study participants

The mean age of participants at death was 91.1 years (SD = 6.1), they had a mean of 14.7 years of education (SD = 2.9), and 71.1% were female, as shown in Table 1 along with other clinical and postmortem characteristics of the sample. The average last valid cognitive score was -0.97 (SD = 0.78), or approximately one standard deviation below the mean at baseline. Proximate to death, participants exhibited mean total daily activity of 1.23 × 10^5^ counts/day (SD = 0.98 × 10^5^), with a relatively wide range from 0.06 × 10^5^ to 4.82 × 10^5^ counts/day. According to Pearson correlation coefficients, total daily physical activity was negatively correlated with age at death (r = -0.24, p < .001) but was not correlated with education (r = -0.04, p = .48) and did not differ between males and females (1.28 vs. 1.22, *t*_316_ = 0.54, p = .59).

**Table 1:** Descriptive characteristics of the sample (N = 318).

Measure	Mean (SD) or n (%)
Clinical measures	
Age at death (years)	91.1 (6.1)
Female	226 (71.1%)
Education (years)	14.7 (2.9)
Total daily physical activity (counts/day ÷ 10^5^)	1.23 (0.98)
Motor abilities (composite of scaled scores)	0.76 (0.19)
Global cognition proximate to death (composite of z-scores)	-0.71 (0.93)
Mini-Mental State Examination (0–30)	23.1 (6.7)
Diagnosis of AD dementia at the time of cognitive testing	117 (36.8%)
Postmortem measures	
Interval from last actigraphy recording to death (years)	1.61 (1.22)
Postmortem interval to neuropathologic examination (hours)	9.1 (7.2)
Neurodegenerative pathology	
Global Alzheimer’s disease pathology (composite of scores)	0.76 (0.59)
Lewy bodies (present)	91 (28.6%)
Hippocampal sclerosis (present)	29 (9.1%)
TAR DNA-binding protein 43 (mod. to severe)	115 (36.2%)
Nigral neuronal loss (mod. to severe)	33 (10.4%)
Cerebrovascular pathology	
Gross infarcts (present)	131 (41.2%)
Microscopic infarcts (present)	99 (31.1%)
Atherosclerosis (mod. to severe)	80 (25.2%)
Arteriolosclerosis (mod. to severe)	89 (28.0%)
Cerebral amyloid angiopathy (mod. to severe)	111 (34.9%)

Participants were older adults from the Rush Memory and Aging Project (MAP). MAP enrollees agree to annual cognitive testing as well as organ donation at death. In this work, we analyzed data from participants who also agreed to wear a wrist-worn activity monitor and for whom we had postmortem brain MRI data available.

### Demographics and cognition

In a base linear regression model with only terms for demographics as predictors, lower education (0.068 unit per year of education, SE = 0.017, p < .001) and older age at death (-0.026 unit per year of age, SE = 0.008, p = .002) were associated with lower global cognition proximate to death. We did not observe a sex difference in cognition (0.16 unit higher in females, SE = 0.11, p = .16).

### Physical activity, brain tissue microstructure (R_2_), and cognition

**Fig 1:**
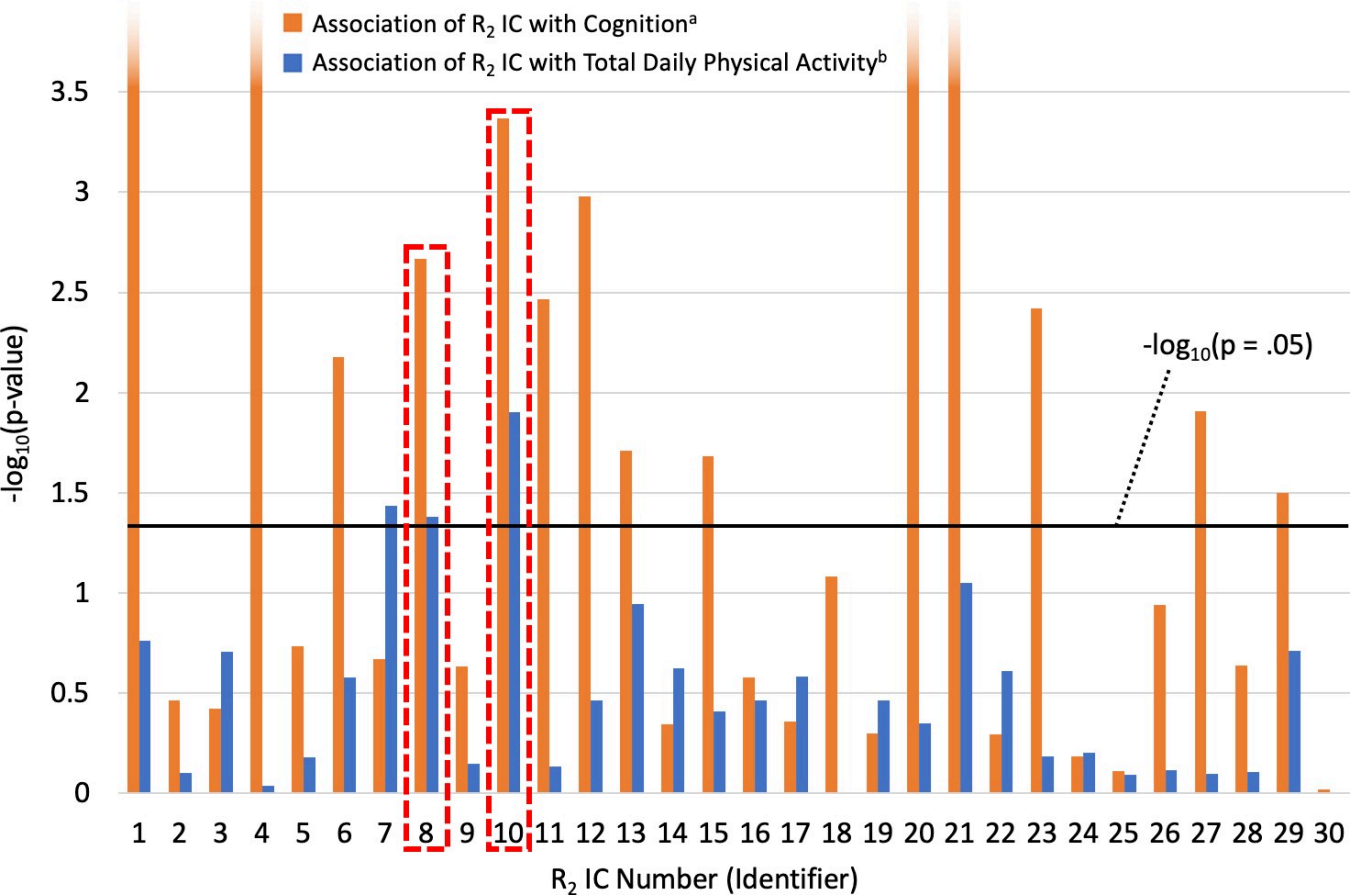
**Identifying brain regions in which tissue microstructure (R**_**2**_**) is associated with both global cognition (orange) and total daily physical activity (blue).** The R_2_ IC identifiers along the horizontal axis refer to the 30 independent components of the transverse relaxation rate, R_2_. Each component captures information on tissue microstructure in a different part of the brain, as illustrated in Fig 2. The plot depicts -log_10_(p-values) on the vertical axis to facilitate side-by-side comparison of the entire range of p-values, including those that are very small (< .00001). Thus, taller bars reflect stronger associations, and some extend beyond the upper limits of what is displayed in this figure. The horizontal black line corresponds to the -log_10_ of p = .05, the nominal significance level we used to screen for candidate R_2_ ICs that might account for the relationship between physical activity and cognition. Of the 30 R_2_ ICs, two (#8 and #10, highlighted in red) were associated with both global cognition and total daily physical activity at p < .05. The linear regression model used to assess the association of R_2_ ICs with global cognition: Global cognition = β_0_ + β_1_*age + β_2_*sex + β_3_*education + β_4_*R_2_, with the p-value of β_4_ plotted in this figure. The linear regression model used to assess the association of total daily physical activity with R_2_ ICs: R_2_ = β_0_ + β_1_*age + β_2_*sex + β_3_*education + β_4_*physical activity, with the p-value of β_4_ plotted in this figure.

**Fig 2:**
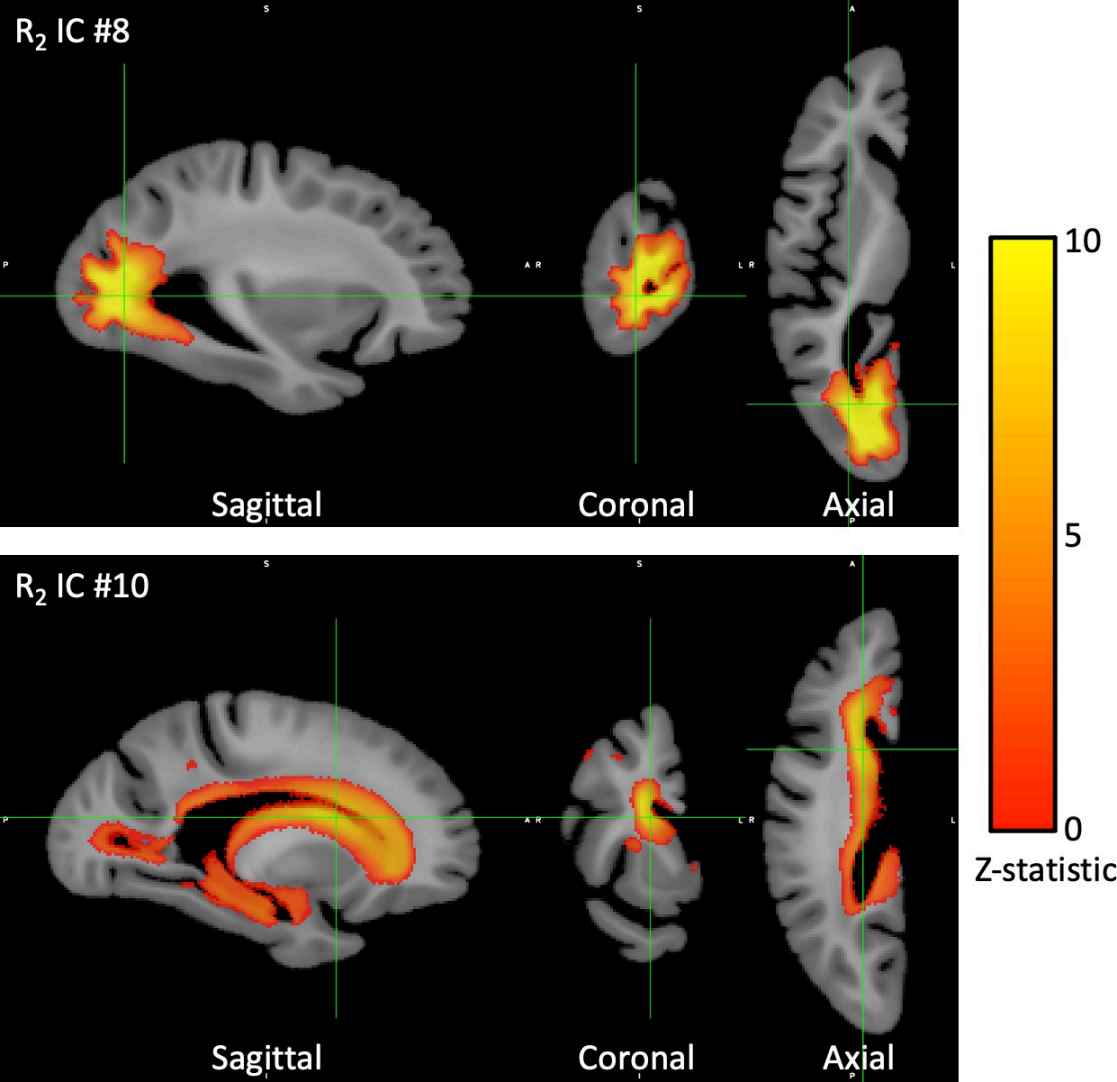
Brain regions in which tissue microstructure (R_2_) partially mediated the association between physical activity and global cognition. Of 30 independent components (ICs), R_2_ IC #8 and #10 were unique in that they were associated with both global cognition and total daily physical activity assessed proximate to death and mediated the association between the two, according to path analysis. This figure highlights the voxels whose R_2_ values contributed strongly to these two ICs. The colorized maps represent the voxelwise Z-statistic output by the FSL MELODIC ICA tool, which reflects the likelihood of a voxel belonging to the active class as opposed to background noise according to mixture modelling (see colorbar at right). For display, we applied a threshold to the IC maps based on a probability value of 0.5 of a voxel belonging to the active class. R_2_ IC #8: White matter and some cortical gray matter portions of the occipital and temporal lobes. R_2_ IC #10: Periventricular white matter extending throughout the frontal, parietal, occipital, and temporal lobes, as well as the hippocampus. The R_2_ values in voxels underlying these regions were negatively associated with both total daily physical activity and cognition; that is, lower R_2_ values (healthier tissue) were linked with higher levels of both total daily physical activity and cognitive function.

Of the 30 ICs derived from ICA of postmortem R_2_ maps of the brain, 14 were associated with global cognition in individual models controlling for age at death, sex, and education, as shown in Fig 1. In a separate set of individual regression models with each of the 30 R_2_ ICs sequentially positioned as the outcome, total daily physical activity was associated with 3 ICs (Fig 1). Comparison of these two sets of screening models revealed that two R_2_ ICs were associated with both physical activity and global cognition and thus might partially account for the relationship between them. One of these ICs (IC #8) mapped chiefly to the white matter of the occipital lobe (Fig 2). The other IC (IC #10) was primarily influenced by the periventricular white matter as well as the hippocampus (Fig 2).

**Fig 3:**
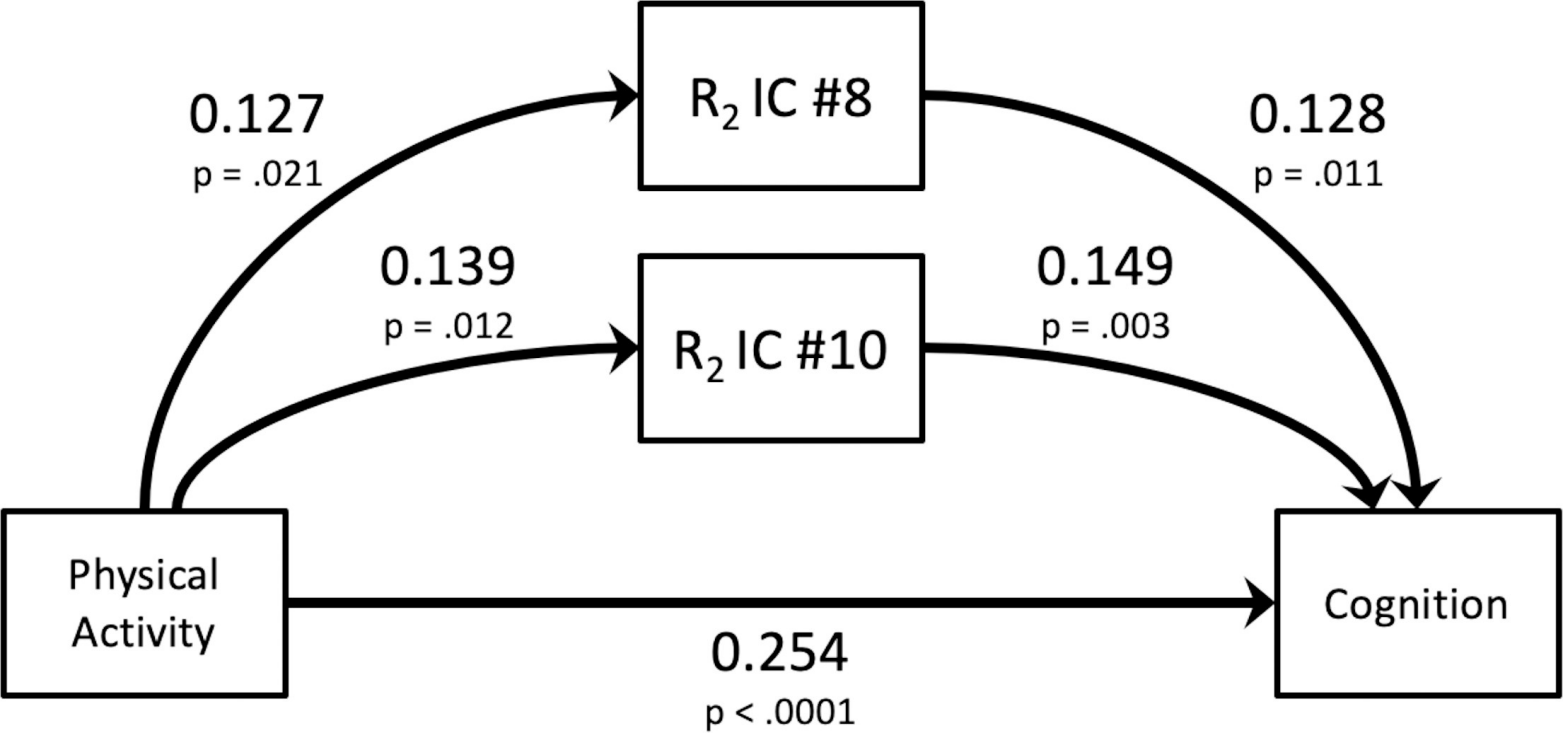
Graphical depiction of path analysis with R_2_ ICs as mediators linking physical activity and cognition. Standardized path coefficients are shown, along with their respective p-values. This analysis also controlled for participants’ age at death, sex, and education (not shown in this figure).

We then conducted path analysis and included IC #8 and IC #10 as potential mediators of the linkage between physical activity and cognition, as illustrated in Fig 3. Both ICs exhibited indirect effects, with standardized path coefficients of 0.016 and 0.021, respectively. In this same path analysis, the standardized coefficient of the direct path between physical activity and cognition was 0.254 (SE = 0.052, p < .001). Thus, the total of direct and indirect path coefficients was 0.291, with the indirect paths via R_2_ IC #8 and IC #10 summing to 0.037 (SE = 0.014, p = .010) and representing 12.7% of the total (Table 2).

**Table 2:** Mediation of the relation between physical activity and global cognition by brain tissue microstructure, in participants with and without AD dementia.

	All Participants (n = 318)		Participants with AD dementia (n = 117)		Participants free of AD dementia (n = 201)	
Effects	Standardized Coefficient (SE)	P-Value	Standardized Coefficient (SE)	P-Value	Standardized Coefficient (SE)	P-Value
Direct path						
Total daily physical activity → Global cognition	0.254 (0.052)	< .001	0.204 (0.090)	.023	0.153 (0.064)	.016
Indirect paths						
Physical activity → R_2_ IC #8	0.127 (0.055)	.021	0.153 (0.091)	.091	0.058 (0.070)	.41
Physical activity → R_2_ IC #10	0.139 (0.055)	.012	-0.000 (0.093)	.99	0.14 (0.069)	.042
R_2_ IC #8 → Global cognition	0.128 (0.050)	.011	0.201 (0.084)	.017	-0.031 (0.063)	.62
R_2_ IC #10 → Global cognition	0.149 (0.050)	.003	-0.109 (0.085)	.20	0.183 (0.063)	.004
Total of indirect paths	0.037 (0.014)	.010	0.031 (0.0246)	.21	0.024 (0.016)	.14
Indirect paths as a percentage of the total	12.7%		13.2%		13.6%	

The Effects column corresponds to the paths between physical activity and global cognition depicted in Fig 3. The direct path is the straight, unmediated linkage between physical activity and cognition. The indirect paths characterize the linkage between physical activity and cognition *via* brain tissue microstructure, R_2_ IC #8 and R_2_ IC #10, which are MRI measures reflecting the tissue microstructure in the occipital lobe and in the periventricular white matter, respectively. The total effect of these indirect paths can be calculated as the sum of the products of the two legs of each indirect path, e.g. for the full 318-person sample, (0.127 × 0.128) + (0.139 × 0.149) = 0.037. The degree to which these indirect paths mediate or account for the total association between physical activity and global cognition can be expressed as a percentage, e.g. for the full 318-person sample, 100 × [0.037/(0.254 + 0.037)] = 12.7%. All models controlled for age, sex, and education.

We also explored whether the relationships among physical activity, R_2_, and global cognition were stronger in participants who were classified as likely having AD dementia compared to those who exhibited no cognitive impairments or were classified as having MCI. In this stratified analysis, R_2_ IC #10 exerted a greater mediating influence than R_2_ IC #8 on the relation between physical activity and global cognition among participants who were free of dementia (Table 2). Conversely, among participant classified as having AD dementia, R_2_ IC #8 was the stronger mediator. However, neither of these mediation effects reached the nominal significance level of .05 according to path analysis.

Substituting each of five cognitive domains as the outcome in the path analysis, the mediating effect of R_2_ on the relation between physical activity and cognition was more prominent in some domains than in others. Most notably, R_2_ IC #8 and R_2_ IC #10 accounted for 17% of the relation of physical activity with episodic memory (p = .014) and 12% of the relation of physical activity with semantic memory (p = .015), as shown in Table 3. The mediating effects of R_2_ did not reach the nominal significance level for semantic memory (p = .085), working memory (p = .070), or visuospatial ability (p = .15).

**Table 3:** Mediation of the relation between physical activity and each of five cognitive domains by brain tissue microstructure.

	Episodic Memory		Semantic Memory		Working Memory		Perceptual Speed		Visuospatial Ability	
Effects	Standardized Coefficient (SE)	P-Value	Standardized Coefficient (SE)	P-Value	Standardized Coefficient (SE)	P-Value	Standardized Coefficient (SE)	P-Value	Standardized Coefficient (SE)	P-Value
Direct path										
Total daily physical activity → Cognition	0.173 (0.054)	.001	0.212 (0.055)	< .001	0.204 (0.055)	< .001	0.244 (0.052)	< .001	0.134 (0.054)	.012
Indirect paths										
Physical activity → R_2_ IC #8	0.122 (0.055)	.028	0.122 (0.055)	.028	0.127 (0.055)	.021	0.123 (0.055)	.027	0.123 (0.055)	.026
Physical activity → R_2_ IC #10	0.141 (0.055)	.010	0.141 (0.055)	.010	0.139 (0.055)	.012	0.148 (0.055)	.007	0.141 (0.055)	.011
R_2_ IC #8 → Cognition	0.085 (0.052)	.10	0.080 (0.053)	.13	0.113 (0.053)	.035	0.111 (0.051)	.029	0.006 (0.052)	.90
R_2_ IC #10 → Cognition	0.181 (0.052)	< .001	0.071 (0.053)	.18	0.056 (0.054)	.30	0.133 (0.051)	.009	0.110 (0.052)	.032
Total of indirect paths	0.036 (0.015)	.014	0.020 (0.012)	.085	0.022 (0.012)	.070	0.033 (0.014)	.015	0.016 (0.011)	.15
Indirect paths as a percentage of the total	17.2%		8.6%		9.7%		11.9%		10.7%	

The Effects column corresponds to the paths between physical activity and global cognition depicted in Fig 3. The direct path is the straight, unmediated linkage between physical activity and cognition. The indirect paths characterize the linkage between physical activity and cognition *via* brain tissue microstructure, R_2_ IC #8 and R_2_ IC #10, which are MRI measures reflecting the tissue microstructure in the occipital lobe and in the periventricular white matter, respectively. These indirect paths were significant mediators of the association between physical activity and cognition for the episodic memory and perceptual speed domains. All models controlled for age, sex, and education.

### Controlling for motor abilities

Because prior work suggests that total daily physical activity is modestly related to motor abilities [55,56] and that both are independently associated with global cognition [7], we repeated the analyses while controlling for a summary measure of motor abilities. As anticipated, motor abilities were associated with global cognition (standardized path coefficient = 0.162, SE = 0.057, p = .005). However, their inclusion in the path analysis did not lead to a substantive change in the indirect effect attributable to R_2_ ICs #8 and #10 in the relationship between total daily physical activity and cognition, which remained at 12.8% (p = .019).

### Controlling for neuropathologic indices

We next repeated the path analysis while controlling for 5 neurodegenerative and 5 cerebrovascular histopathologic indices, given that some of these are associated with global cognition in late life as well as with R_2_ measured in postmortem brain [31]. In these models, the indirect effect via the two R_2_ ICs ranged from 9.9% (p = .014) when controlling for the global measure of AD pathology to 15.1% (p = .007) when controlling for nigral neuronal loss. In a combined model controlling for all 10 pathologies simultaneously, the indirect effect via IC #8 and #10 was 12.9% (p = .009).

### Controlling for other potential confounders

We additionally repeated the path analysis while controlling for other potential confounders, including chronic medical conditions, vascular disease and risk factors, depression, and body mass index. The number of chronic medical conditions was associated with cognition, but this did not appreciably alter the apparent mediation by R_2_ ICs #8 and #10. A sensitivity analysis also showed that the apparent mediation of the linkage between physical activity and cognition by R_2_ was essentially unchanged when we excluded 17 cases for which the lag between the cognitive exam and autopsy was greater than two years.

## Discussion

Based on clinical and postmortem data from more than 300 community-dwelling older adults, our analyses are consistent with the notion that brain tissue microstructure, as quantified by postmortem MRI, partially mediates the association between higher levels of physical activity and better cognitive function. This partial mediation remained evident even after we controlled for motor abilities and common age-related brain pathologies, including both neurodegenerative and cerebrovascular pathologies. Furthermore, the effect was observed in participants with and without AD dementia. Thus, this work adds to a growing literature suggesting that some lifestyle factors are associated with cognition via measurable biologic factors other than neuropathologies with well-documented links to dementia. In particular, the current findings indicate that a more active lifestyle in older adults may confer cognitive benefits in part through a neurobiologic pathway involving brain tissue microstructure that can be quantified and visualized using MRI. These findings may help to direct future studies focused on specific brain regions to elucidate the molecular mechanisms underlying this pathway, thereby catalyzing further drug discovery for maintaining cognition. More generally, this study highlights the means by which postmortem brain imaging can be used to identify possible sources of brain reserve that are associated with different behavioral phenotypes in aging adults.

Results of this work are broadly consistent with prior research but also extend our understanding of the relationships among physical activity, brain microstructure, and cognition. First, the well-established association between higher levels of physical activity and better cognition in late life [57] was indeed borne out in the current study. Elucidating the neurobiologic link between this modifiable lifestyle factor and cognition has garnered much research focus in recent years. The result of these efforts is a large body of work that has revealed a link between physical activity and MRI-based measures of brain tissue microstructure in older adults [11,12], complemented by other studies in humans and animals showing that exercise-related up-regulations of neurotrophic factors such as BDNF, IGF-1, and VEGF likely contribute to improved microstructural integrity of brain tissue [58–60]. In addition, studies have identified MRI signatures that are associated with cognitive abilities [33] rate of cognitive decline [61], and AD dementia among older adults [62]. However, few studies have investigated the relationships among the triad of quantitative metrics of total daily physical activity, MRI-based measures of brain tissue microstructure, and cognition in the same older adults [8,18–20]. Accordingly, one novel contribution of this study is the quantification of the portion of the association between physical activity and late life cognition that can be accounted for by MRI measures of brain tissue microstructure, which came to approximately 12.7%. This finding lends support to the hypothesis that brain tissue microstructure partially mediates the relationship between physical activity and cognition.

Further insight regarding the physical meaning and clinical implications of the partial mediation can be gained by examining the directionality of the associations among physical activity, MRI-based measures of brain tissue microstructure (i.e. the R_2_ ICs), and cognition. Of note, prior work showed that faster transverse relaxation (i.e. higher R_2_) in white matter, likely reflecting lower free water content related to healthier tissue microstructure, was associated with better cognitive function (i.e. higher cognitive scores) and shallower trajectories of decline (i.e. less negative rates of decline) [33,61]. This is consistent with observations in the current study. Namely, the R_2_ values underlying IC #8 and #10 were each positively associated with cognition proximate to death. The associations between total daily physical activity and these R_2_ ICs were also positive, meaning that higher levels of physical activity were associated with higher R_2_ values. Taken together, a plausible interpretation of these results is that a portion of the cognitive benefits related to high levels of physical activity are conferred via maintenance or improvements to brain tissue microstructure, which are reflected in higher values of R_2_ in certain key regions consisting mainly of white matter. Based on this hypothesis, future studies examining MRI and cognitive data collected at multiple timepoints during life might help to answer the potentially important question of whether activity-related improvements to the white matter’s microstructural integrity represent a source of brain reserve, i.e., one’s capacity to maintain cognitive function, despite the onset of disease processes such as Alzheimer’s pathophysiology.

The spatial signatures of R_2_ ICs #8 and #10 can potentially direct the study of molecular mechanisms underlying the association between physical activity and cognition in specific brain regions. These signatures share certain similarities with regions previously shown to have associations between R_2_ and cognition, but also differ in important ways. Notably, in prior studies, large portions of the white matter in the frontal and temporal lobes were the regions most robustly associated with cognition [31,38,61]. The current work is consistent with those findings; IC #1 and IC #4, whose spatial signatures covered frontal and temporal lobe white matter, respectively, were the two ICs most strongly associated with cognition proximate to death. However, IC #1 and IC #4 were not associated with total daily physical activity. Instead, when it comes to the relationship between physical activity and cognition, our results suggest that periventricular white matter underlying R_2_ IC #10 may have a more potent mediating influence. Interestingly, IC #10 bears a striking resemblance to the pattern of periventricular hyperintensities commonly observed on T_2_-weighted and FLAIR MRI of older adults’ brains, which are increasingly recognized as having clinical implications for cognition [63]. R_2_ IC #8 primarily encompassed the white matter of the occipital lobe, with some extension into the posterior portion of the temporal lobe. The fact that path analysis showed IC #8 as partially mediating the relationship between physical activity and cognition was unexpected because to our knowledge, tissue integrity or damage in the occipital lobe has not previously been highlighted as a particularly strong correlate of physical activity. However, in light of this region’s importance in the visual system, it might be worthwhile in future studies to examine whether the type or quantity of visual stimuli inherent to an active lifestyle might play a role in the link between physical activity and cognition.

We also examined which cognitive domains were most strongly linked to physical activity via a pathway involving R_2_. For episodic memory, R_2_ IC #8 and R_2_ IC #10 together accounted for 17% of the relation between physical activity and cognition, or about 4 percentage points higher than what was observed for global cognition. Episodic memory impairment is one of the defining features of AD dementia. Thus, this domain-specific finding is an additional piece of evidence suggesting that physical activity might be important for maintenance of brain health, which in turn offers resilience against AD dementia. R_2_ IC #8 and R_2_ IC #10 also accounted for 12% of the relation between physical activity and the perceptual speed domain, about the same as what was observed for global cognition. In earlier reports, white matter was linked to processing speed in healthy young people [64], and in the current work, both IC #8 and #10 encompassed significant portions of white matter. This hints at one possible explanation for the mediating effect of R_2_, whereby high levels of physical activity in older age also reflect a lifelong pattern of physical activity that helps to promote white matter integrity and better perceptual speed.

Stratified analyses suggest that R_2_ IC #10 is the more potent mediator in people without AD dementia, while R_2_ IC #8 is more involved in people who have been diagnosed with dementia. Neither of these mediation effects reached the level of statistical significance, perhaps owing to the reduced power in the stratified analyses compared to the main analysis. However, these results hint at the possibility that certain regions of the brain might offer stronger resilience against cognitive decline at certain periods during the progression of disease.

We accounted for the likely involvement of motor abilities due to their previously reported correlations with physical activity level [55,56,65]. As in earlier work, we found in the current study that better motor abilities were indeed associated with better cognitive function proximate to death [7]. Nevertheless, the mediating effect of R_2_ ICs #8 and #10 on the association between physical activity and cognition remained almost unchanged, with an indirect effect of 12.8% after controlling for motor abilities. The implication is that at least a portion of the neurobiologic pathway linking physical activity to cognition might not depend on motor abilities. In other words, a high level of physical activity may help to promote brain health and cognition to some extent, regardless of one’s level of motor abilities.

In prior work, we examined histopathologic indices of neurodegenerative and cerebrovascular pathologies and found no convincing evidence that these pathologies were involved in the relationship between physical activity and cognition in older adults [7]. The R_2_ IC measures of the current work may be regarded as another type of index that probe tissue microstructure throughout the cerebral hemisphere, and our analyses showed that they did have a mediating influence on the relationship between physical activity and cognition. Furthermore, controlling for available neuropathologic indices did not reduce the magnitude of this mediation. These findings suggest that the MRI-derived R_2_ metrics capture unique information regarding the neurobiologic basis of cognitive benefits related to physical activity in late life.

This study has notable strengths and limitations. One major strength lies in the approach of collecting objective measures of physical activity and cognition in the years just prior to death, which facilitated linking these data with corresponding postmortem MRI and histopathologic indices. It is otherwise difficult to align imaging and clinical measures in large numbers of older participants, who may be unable or unwilling to travel to an MRI site to undergo imaging. The availability of postmortem brain MRI from large numbers of well-characterized older adults is another major strength of this work, but one side effect is that collection of this data necessarily occurred over the span of several years, using different models of 3-Tesla scanners. Combining data from multiple scanners was a concern, but we took steps to normalize these data and thereby minimize the chance of scanner-related bias affecting the results. We also note that R_2_ values decrease substantially after death and chemical fixation [32]. Therefore, additional study will be required in order to translate the current findings to the in vivo case. There are also certain limitations inherent to this study’s approach, some of which stem from the fact that we examined cross-sectional data from an observational study; firm conclusions regarding the direction of causality cannot be established by such studies. Our *a priori* assumption was that physical activity resides upstream of tissue microstructure (as captured by R_2_ imaging metrics) and cognition in the causal chain, but these factors likely influence each other to some extent. Future investigations of physical activity-based interventions are necessary to provide empiric support for a causal relationship between physical activity, brain structure, and improved cognition in older adults. Another limitation of the study lies in the fact that MAP participants are selected and tend to be more highly educated, which may contribute to higher levels of physical activity and differences in other key behaviors and experiential factors compared to the general population. Studies in broader samples are therefore needed to extend the clinical relevance of the study’s findings. While we did not explicitly control for multiple comparisons in these analyses, the two-stage process whereby we first screened for R_2_ components before analyzing just two in path analysis likely provided adequate protection against the risk of spurious findings. Furthermore, results of path analysis were essentially unchanged when we controlled for a variety of potentially confounding factors, lending additional confidence to our main findings. Although we accounted for several types of cerebrovascular pathologies, it is possible that additional vascular lesions remain unquantified by either histology or 3-Tesla MRI. Use of higher field strength MRI might capture lesions such as microbleeds more fully, which would help to enhance our understanding of the relationships among physical activity, brain pathology detected via histology and MRI, and cognition. It is possible that wearing the activity monitor influenced participants to alter their activity patterns. Therefore, we must interpret this study’s results while bearing in mind that the total daily physical activity measure might include some amount of extra activity above and beyond usual activity levels due to participants’ knowledge of what the device captures.

## References

[1] Phillips C. Lifestyle modulators of neuroplasticity: how physical activity, mental engagement, and diet promote cognitive health during aging. Neural Plast. 2017;2017. 10.1155/2017/3589271 PMC5485368 28695017

[2] Livingston G, Sommerlad A, Orgeta V, Costafreda SG, Huntley J, Ames D, et al. Dementia prevention, intervention, and care. The Lancet. 2017;390: 2673–2734. 10.1016/S0140-6736(17)31363-6 28735855

[3] Liu T, Luo H, Tang JY, Wong GH. Does lifestyle matter? Individual lifestyle factors and their additive effects associated with cognitive function in older men and women. Aging & Mental Health. 2020;24: 405–412. doi: 10.1080/13607863.2018.1539833 30520690

[4] Clare L, Wu Y, Teale JC, MacLeod C, Matthews F, Brayne C, et al. Potentially modifiable lifestyle factors, cognitive reserve, and cognitive function in later life: A cross-sectional study. PLoS medicine. 2017;14. 10.1371/journal.pmed.1002259 PMC5360216 28323829

[5] Blondell SJ, Hammersley-Mather R, Veerman JL. Does physical activity prevent cognitive decline and dementia?: A systematic review and meta-analysis of longitudinal studies. BMC Public Health. 2014;14: 510. doi: 10.1186/1471-2458-14-510 24885250 PMC4064273

[6] Buchman AS, Boyle PA, Yu L, Shah RC, Wilson RS, Bennett DA. Total daily physical activity and the risk of AD and cognitive decline in older adults. Neurology. 2012;78: 1323–1329. doi: 10.1212/WNL.0b013e3182535d35 22517108 PMC3335448

[7] Buchman AS, Yu L, Wilson RS, Lim A, Dawe RJ, Gaiteri C, et al. Physical activity, common brain pathologies, and cognition in community-dwelling older adults. Neurology. 2019;92: e811–e822. doi: 10.1212/WNL.0000000000006954 30651386 PMC6396972

[8] Voss MW, Heo S, Prakash RS, Erickson KI, Alves H, Chaddock L, et al. The influence of aerobic fitness on cerebral white matter integrity and cognitive function in older adults: Results of a one‐year exercise intervention. Hum Brain Mapp. 2013;34: 2972–2985. doi: 10.1002/hbm.22119 22674729 PMC4096122

[9] Xiao Q, Wang F, Luo Y, Chen L, Chao F, Tan C, et al. Exercise protects myelinated fibers of white matter in a rat model of depression. J Comp Neurol. 2018;526: 537–549. doi: 10.1002/cne.24350 29098693

[10] Chao F, Zhang L, Zhang Y, Zhou C, Jiang L, Xiao Q, et al. Running exercise protects against myelin breakdown in the absence of neurogenesis in the hippocampus of AD mice. Brain Res. 2018;1684: 50–59. doi: 10.1016/j.brainres.2018.01.007 29317290

[11] Burzynska AZ, Chaddock-Heyman L, Voss MW, Wong CN, Gothe NP, Olson EA, et al. Physical activity and cardiorespiratory fitness are beneficial for white matter in low-fit older adults. PloS one. 2014;9: e107413. doi: 10.1371/journal.pone.0107413 25229455 PMC4167864

[12] Johnson NF, Kim C, Clasey JL, Bailey A, Gold BT. Cardiorespiratory fitness is positively correlated with cerebral white matter integrity in healthy seniors. Neuroimage. 2012;59: 1514–1523. doi: 10.1016/j.neuroimage.2011.08.032 21875674 PMC3230672

[13] Arvanitakis Z, Fleischman DA, Arfanakis K, Leurgans SE, Barnes LL, Bennett DA. Association of white matter hyperintensities and gray matter volume with cognition in older individuals without cognitive impairment. Brain Structure and Function. 2016;221: 2135–2146. doi: 10.1007/s00429-015-1034-7 25833685 PMC4592368

[14] Cremers LG, de Groot M, Hofman A, Krestin GP, van der Lugt A, Niessen WJ, et al. Altered tract-specific white matter microstructure is related to poorer cognitive performance: the Rotterdam Study. Neurobiol Aging. 2016;39: 108–117. doi: 10.1016/j.neurobiolaging.2015.11.021 26923407

[15] Li Y, Liu Y, Li J, Qin W, Li K, Yu C, et al. Brain anatomical network and intelligence. PLoS Comput Biol. 2009;5: e1395. doi: 10.1371/journal.pcbi.1000395 19492086 PMC2683575

[16] Fischer FU, Wolf D, Tüscher O, Fellgiebel A, Alzheimer’s Disease Neuroimaging Initiative. Structural Network Efficiency Predicts Resilience to Cognitive Decline in Elderly at Risk for Alzheimer’s Disease. Frontiers in aging neuroscience. 2021;13: 44. doi: 10.3389/fnagi.2021.637002 33692682 PMC7937862

[17] Reuter-Lorenz PA, Park DC. How does it STAC up? Revisiting the scaffolding theory of aging and cognition. Neuropsychol Rev. 2014;24: 355–370. doi: 10.1007/s11065-014-9270-9 25143069 PMC4150993

[18] Tian Q, Glynn NW, Erickson KI, Aizenstein HJ, Simonsick EM, Yaffe K, et al. Objective measures of physical activity, white matter integrity and cognitive status in adults over age 80. Behav Brain Res. 2015;284: 51–57. doi: 10.1016/j.bbr.2015.01.045 25655514 PMC4369426

[19] Oberlin LE, Verstynen TD, Burzynska AZ, Voss MW, Prakash RS, Chaddock-Heyman L, et al. White matter microstructure mediates the relationship between cardiorespiratory fitness and spatial working memory in older adults. Neuroimage. 2016;131: 91–101. doi: 10.1016/j.neuroimage.2015.09.053 26439513 PMC4826637

[20] Stillman CM, Cohen J, Lehman ME, Erickson KI. Mediators of physical activity on neurocognitive function: a review at multiple levels of analysis. Frontiers in human neuroscience. 2016;10: 626. doi: 10.3389/fnhum.2016.00626 28018195 PMC5161022

[21] Bennett DA, Buchman AS, Boyle PA, Barnes LL, Wilson RS, Schneider JA. Religious Orders Study and Rush Memory and Aging Project. J Alzheimer’s Dis. 2018: 1–28. doi: 10.3233/JAD-179939 29865057 PMC6380522

[22] Buchman AS, Yu L, Boyle PA, Shah RC, Bennett DA. Total daily physical activity and longevity in old age. Arch Intern Med. 2012;172: 444–446. doi: 10.1001/archinternmed.2011.1477 22412115 PMC3366177

[23] Heil DP. Predicting activity energy expenditure using the Actical® activity monitor. Res Q Exerc Sport. 2006;77: 64–80. doi: 10.1080/02701367.2006.10599333 16646354

[24] Wilson RS, Segawa E, Boyle PA, Anagnos SE, Hizel LP, Bennett DA. The natural history of cognitive decline in Alzheimer’s disease. Psychol Aging. 2012;27: 1008. doi: 10.1037/a0029857 22946521 PMC3534850

[25] Boyle PA, Wilson RS, Yu L, Barr AM, Honer WG, Schneider JA, et al. Much of late life cognitive decline is not due to common neurodegenerative pathologies. Ann Neurol. 2013;74: 478–489. doi: 10.1002/ana.23964 23798485 PMC3845973

[26] Wilson RS, Beckett LA, Barnes LL, Schneider JA, Bach J, Evans DA, et al. Individual differences in rates of change in cognitive abilities of older persons. Psychol Aging. 2002;17: 179. 12061405

[27] Bennett DA, Schneider JA, Buchman AS, de Leon CM, Bienias JL, Wilson RS. The Rush Memory and Aging Project: study design and baseline characteristics of the study cohort. Neuroepidemiology. 2005;25: 163–175. doi: 10.1159/000087446 16103727

[28] Bennett DA, Schneider JA, Aggarwal NT, Arvanitakis Z, Shah RC, Kelly JF, et al. Decision rules guiding the clinical diagnosis of Alzheimer’s disease in two community-based cohort studies compared to standard practice in a clinic-based cohort study. Neuroepidemiology. 2006;27: 169–176. doi: 10.1159/000096129 17035694

[29] Bennett DA, Wilson RS, Schneider JA, Evans DA, Beckett LA, Aggarwal NT, et al. Natural history of mild cognitive impairment in older persons. Neurology. 2002;59: 198–205. doi: 10.1212/wnl.59.2.198 12136057

[30] Buchman AS, Leurgans SE, Yu L, Wilson RS, Lim AS, James BD, et al. Incident parkinsonism in older adults without Parkinson disease. Neurology. 2016;87: 1036–1044. doi: 10.1212/WNL.0000000000003059 27488597 PMC5027813

[31] Dawe RJ, Bennett DA, Schneider JA, Leurgans SE, Kotrotsou A, Boyle PA, et al. Ex vivo T2 relaxation: associations with age-related neuropathology and cognition. Neurobiol Aging. 2014;35: 1549–1561. doi: 10.1016/j.neurobiolaging.2014.01.144 24582637 PMC3989898

[32] Dawe RJ, Bennett DA, Schneider JA, Vasireddi SK, Arfanakis K. Postmortem MRI of human brain hemispheres: T2 relaxation times during formaldehyde fixation. Magn Reson Med. 2009;61: 810–818. doi: 10.1002/mrm.21909 19189294 PMC2713761

[33] Dawe RJ, Bennett DA, Schneider JA, Leurgans SE, Kotrotsou A, Boyle PA, et al. Ex vivo T 2 relaxation: associations with age-related neuropathology and cognition. Neurobiol Aging. 2014;35: 1549–1561. doi: 10.1016/j.neurobiolaging.2014.01.144 24582637 PMC3989898

[34] Laule C, Leung E, Li DK, Traboulsee AL, Paty DW, MacKay AL, et al. Myelin water imaging in multiple sclerosis: quantitative correlations with histopathology. Multiple Sclerosis Journal. 2006;12: 747–753. doi: 10.1177/1352458506070928 17263002

[35] House MJ, St. Pierre TG, McLean C. 1.4 T study of proton magnetic relaxation rates, iron concentrations, and plaque burden in Alzheimer’s disease and control postmortem brain tissue. Magnetic Resonance in Medicine: An Official Journal of the International Society for Magnetic Resonance in Medicine. 2008;60: 41–52. 10.1002/mrm.21586 18523986

[36] Gudbjartsson H, Patz S. The Rician distribution of noisy MRI data. Magnetic resonance in medicine. 1995;34: 910–914. doi: 10.1002/mrm.1910340618 8598820 PMC2254141

[37] Avants BB, Tustison N, Song G. Advanced normalization tools (ANTS). Insight j. 2009;2: 1–35.

[38] Dawe RJ, Yu L, Schneider JA, Arfanakis K, Bennett DA, Boyle PA. Postmortem Brain MRI Is Related to Cognitive Decline, Independent of Cerebral Vessel Disease in Older Adults. Neurobiol Aging. 2018. doi: 10.1016/j.neurobiolaging.2018.05.020 29908416 PMC6424332

[39] Beckmann CF, Smith SM. Probabilistic independent component analysis for functional magnetic resonance imaging. IEEE Trans Med Imaging. 2004;23: 137–152. doi: 10.1109/TMI.2003.822821 14964560

[40] Smith SM, Brady JM. SUSAN—a new approach to low level image processing. International journal of computer vision. 1997;23: 45–78.

[41] Willette AA, Calhoun VD, Egan JM, Kapogiannis D, Alzheimer׳ s Disease Neuroimaging Initiative. Prognostic classification of mild cognitive impairment and Alzheimer׳ s disease: MRI independent component analysis. Psychiatry Research: Neuroimaging. 2014;224: 81–88. doi: 10.1016/j.pscychresns.2014.08.005 25194437 PMC4586157

[42] Pagani M, Giuliani A, Öberg J, De Carli F, Morbelli S, Girtler N, et al. Progressive disintegration of brain networking from normal aging to Alzheimer disease: analysis of independent components of 18F-FDG PET data. Journal of Nuclear Medicine. 2017;58: 1132–1139. doi: 10.2967/jnumed.116.184309 28280223

[43] Caprihan A, Abbott C, Yamamoto J, Pearlson G, Perrone-Bizzozero N, Sui J, et al. Source-based morphometry analysis of group differences in fractional anisotropy in schizophrenia. Brain connectivity. 2011;1: 133–145. doi: 10.1089/brain.2011.0015 22180852 PMC3236525

[44] Xu L, Groth KM, Pearlson G, Schretlen DJ, Calhoun VD. Source-based morphometry: The use of independent component analysis to identify gray matter differences with application to schizophrenia. Hum Brain Mapp. 2009;30: 711–724. doi: 10.1002/hbm.20540 18266214 PMC2751641

[45] Liu K, Yao S, Chen K, Zhang J, Yao L, Li K, et al. Structural brain network changes across the adult lifespan. Frontiers in aging neuroscience. 2017;9: 275. doi: 10.3389/fnagi.2017.00275 28860988 PMC5562685

[46] Bennett DA, Schneider JA, Buchman AS, Barnes LL, Boyle PA, Wilson RS. Overview and findings from the rush Memory and Aging Project. Current Alzheimer Research. 2012;9: 646–663. doi: 10.2174/156720512801322663 22471867 PMC3439198

[47] Schneider JA, Li J, Li Y, Wilson RS, Kordower JH, Bennett DA. Substantia nigra tangles are related to gait impairment in older persons. Ann Neurol. 2006;59: 166–173. doi: 10.1002/ana.20723 16374822

[48] Schneider JA, Arvanitakis Z, Yu L, Boyle PA, Leurgans SE, Bennett DA. Cognitive impairment, decline and fluctuations in older community-dwelling subjects with Lewy bodies. Brain. 2012;135: 3005–3014. doi: 10.1093/brain/aws234 23065790 PMC3470712

[49] Nag S, Yu L, Capuano AW, Wilson RS, Leurgans SE, Bennett DA, et al. Hippocampal sclerosis and TDP‐43 pathology in aging and A lzheimer disease. Ann Neurol. 2015;77: 942–952. doi: 10.1002/ana.24388 25707479 PMC4447563

[50] Arvanitakis Z, Leurgans SE, Wang Z, Wilson RS, Bennett DA, Schneider JA. Cerebral amyloid angiopathy pathology and cognitive domains in older persons. Ann Neurol. 2011;69: 320–327. doi: 10.1002/ana.22112 21387377 PMC3228518

[51] Schneider JA, Bienias JL, Wilson RS, Berry-Kravis E, Evans DA, Bennett DA. The apolipoprotein E ε4 allele increases the odds of chronic cerebral infarction detected at autopsy in older persons. Stroke. 2005;36: 954–959. doi: 10.1161/01.STR.0000160747.27470.2a 15774818

[52] Arvanitakis Z, Capuano AW, Leurgans SE, Buchman AS, Bennett DA, Schneider JA. The relationship of cerebral vessel pathology to brain microinfarcts. Brain Pathol. 2017;27: 77–85. doi: 10.1111/bpa.12365 26844934 PMC4974145

[53] Buchman AS, Leurgans SE, Nag S, Bennett DA, Schneider JA. Cerebrovascular disease pathology and parkinsonian signs in old age. Stroke. 2011;42: 3183–3189. doi: 10.1161/STROKEAHA.111.623462 21885844 PMC3202031

[54] Boyle PA, Yu L, Nag S, Leurgans S, Wilson RS, Bennett DA, et al. Cerebral amyloid angiopathy and cognitive outcomes in community-based older persons. Neurology. 2015;85: 1930–1936. doi: 10.1212/WNL.0000000000002175 26537052 PMC4664125

[55] Dawe RJ, Leurgans SE, Yang J, Bennett JM, Hausdorff JM, Lim AS, et al. Association between quantitative gait and balance measures and total daily physical activity in community-dwelling older adults. The Journals of Gerontology: Series A. 2017;73: 636–642. 10.1093/gerona/glx167 PMC5905609 28957994

[56] James BD, Boyle PA, Bennett DA, Buchman AS. Total daily activity measured with actigraphy and motor function in community-dwelling older persons with and without dementia. Alzheimer Dis Assoc Disord. 2012;26: 238. doi: 10.1097/WAD.0b013e31822fc3cb 21946015 PMC3251727

[57] Brown BM, Peiffer JJ, Martins RN. Multiple effects of physical activity on molecular and cognitive signs of brain aging: can exercise slow neurodegeneration and delay Alzheimer’s disease? Mol Psychiatry. 2013;18: 864. doi: 10.1038/mp.2012.162 23164816

[58] Leckie RL, Oberlin LE, Voss MW, Prakash RS, Szabo-Reed A, Chaddock-Heyman L, et al. BDNF mediates improvements in executive function following a 1-year exercise intervention. Frontiers in human neuroscience. 2014;8: 985. doi: 10.3389/fnhum.2014.00985 25566019 PMC4263078

[59] Cassilhas RC, Antunes HKM, Tufik S, De Mello MT. Mood, anxiety, and serum IGF-1 in elderly men given 24 weeks of high resistance exercise. Percept Mot Skills. 2010;110: 265–276. doi: 10.2466/PMS.110.1.265-276 20391891

[60] Kraus RM, Stallings HW III, Yeager RC, Gavin TP. Circulating plasma VEGF response to exercise in sedentary and endurance-trained men. J Appl Physiol. 2004;96: 1445–1450. doi: 10.1152/japplphysiol.01031.2003 14660505

[61] Dawe RJ, Yu L, Leurgans SE, Schneider JA, Buchman AS, Arfanakis K, et al. Postmortem MRI: a novel window into the neurobiology of late life cognitive decline. Neurobiol Aging. 2016;45: 169–177. doi: 10.1016/j.neurobiolaging.2016.05.023 27459937 PMC5003419

[62] Yu L, Dawe RJ, Buchman AS, Boyle PA, Schneider JA, Arfanakis K, et al. Ex vivo MRI transverse relaxation in community based older persons with and without Alzheimer’s dementia. Behav Brain Res. 2017;322: 233–240. doi: 10.1016/j.bbr.2016.09.001 27596378 PMC5325779

[63] Griffanti L, Jenkinson M, Suri S, Zsoldos E, Mahmood A, Filippini N, et al. Classification and characterization of periventricular and deep white matter hyperintensities on MRI: a study in older adults. Neuroimage. 2018;170: 174–181. doi: 10.1016/j.neuroimage.2017.03.024 28315460

[64] Magistro D, Takeuchi H, Nejad KK, Taki Y, Sekiguchi A, Nouchi R, et al. The relationship between processing speed and regional white matter volume in healthy young people. PloS one. 2015;10: e0136386. doi: 10.1371/journal.pone.0136386 26397946 PMC4580478

[65] Morie M, Reid KF, Miciek R, Lajevardi N, Choong K, Krasnoff JB, et al. Habitual physical activity levels are associated with performance in measures of physical function and mobility in older men. J Am Geriatr Soc. 2010;58: 1727–1733. doi: 10.1111/j.1532-5415.2010.03012.x 20738436 PMC2945416

